# MicroRNA-181b regulates endotoxin tolerance by targeting IL-6 in macrophage RAW264.7 cells

**DOI:** 10.1186/s12950-015-0061-8

**Published:** 2015-03-02

**Authors:** Wenjun Zhang, Xiaojun Shen, Luyang Xie, Maoping Chu, Yanmei Ma

**Affiliations:** Department of Emergency, Changhai Hospital, Shanghai, 200433 China; Children’s Heart Center, the Second Affiliated Hospital & Yuying Children’s Hospital, Institute of Cardiovascular Development and Translation Medicine, Wenzhou Medical University, Wenzhou, 325000 Zhejiang Province China; Department of General Surgery, Changhai Hospital, Shanghai, 200433 China; Department of Stomatology, Shanghai Tenth People’s Hospital, Shanghai, 200072 China

**Keywords:** miR-181b, IL-6, Endotoxin tolerance, LPS, Macrophage

## Abstract

Interleukin 6 (IL-6) is a major pro-inflammatory cytokine and dysregulation of IL-6 is relevant to many inflammatory diseases. Endotoxin induced tolerance of IL-6 is an important mechanism to avoid the excessive immune reaction. But to date, the molecular mechanisms of endotoxin tolerance of IL-6 remain unclear. Here we reported that IL-6 secretion and microRNA-181b (miR-181b) expression were inversely correlated following LPS stimulation. We also demonstrated that miR-181b targeting the 3′-UTR of IL-6 transcripts and up-regulation of miR-181b was associated with NF-kB. We further demonstrated that up-regulation of miR-181b in response to LPS was required for inducing IL-6 tolerance in macrophage. Our results suggested that the post-transcriptional control mediated by miR-181b could be involved in fine tuning the critical level of IL-6 expression in endotoxin tolerance.

## Background

The innate immune system is an evolutionarily conserved defense mechanism against pathogens, which have been detected by the fundamental pattern recognition receptors (PRRs). Toll like receptors (TLRs) family have emerged as an important group of PRRs [1]. During bacterial, viral, and fungal invasion, danger signals are effectively detected by TLR4 and lead to inflammatory cells activation, pro-inflammatory cytokines/chemokines production (e.g., TNF-a, IL-1, IL-6 and COX-2) through the MyD88–dependent and/or MyD88-independent signaling pathway. And then, after the burst of pro-inflammatory cytokines, the body developed a compensatory anti-inflammatory response and released anti-inflammatory cytokines such as IL-10 and TGF-β that inhibit the pro-inflammatory immune response [2]. These inflammatory reactions which induced by LPS are necessary for neutralizing the expand of gram-negative bacteria, but it should be tightly regulated *in vivo*. Uncontrolled inflammation leads to extensive tissue damage, sepsis syndrome, and even endotoxin shock [3]. The immune system has developed a defence mechanism including endotoxin tolerance against these uncontrolled inflammations thus preventing harmful pathologies like sepsis.

IL-6 is one important pro-inflammatory cytokines in inflammatory response and must be down-regulated in order to avoid excessive inflammation [4]. LPS tolerance was reduced in SOCS-1 deficient mice as shown by increasing TNF-α and IL-6 production which compared with the wild-type mice [5]. Activation of PPAR gamma by LPS negatively regulates macrophage activation by decreasing TNF-α, IL-6, IL-1β and NO production [6]. Similarly, ATF3 is reported to down-regulate IL-6 production and protect from LPS shock in mice [7]. But to date, the molecular mechanisms of endotoxin tolerance of IL-6 remain to be resolved clearly.

MicroRNAs (miRNAs) are one small noncoding RNAs which regulate gene expression by targeting the 3′-untranslated regions (3′-UTR) of target mRNAs, and have diverse functional roles in biological processes [8]. The endogenously expressed miRNAs have been identified as important contributors to many physiological and pathological processes including immune tolerance [9]. miRNAs can regulate tolerance at many levels of the cell signaling cascade. The roles such as miR-146a, miR-221, miR-579, miR-125b, miR-155, let-7e, and miR-98 have been identified in regulating the TLR4 signaling pathway during the development of endotoxin tolerance at receptor, signaling pathway, gene transcription and translational levels [10]. However, it remains unclear whether miRNA mediated regulatory mechanism is involved in IL-6 production in TLR-triggered macrophages tolerance.

In the present study, we reported that up-regulation of miR-181b after LPS exposure partially contributes to down-regulation of IL-6, which might be an important regulatory mechanism for controlling pro-inflammatory immune response and induction of endotoxin tolerance.

## Methods

### Cell culture and tolerance

HEK-293 and RAW264.7 cell lines were obtained from American Type Culture Collection (Manassas, VA) and cultured in DMEM containing 10% FBS. Cells were maintained at 37.0°C in a humidified atmosphere with 5% CO_2_.

RAW264.7 cells were made tolerant by incubation with 100 ng/ml LPS (Sigma-Aldrich, St. Louis, MO). 24 h later, tolerant cells were washed and resuspended in complete medium.

### Luciferase reporter assays

Luciferase reporter construct was made by cloning human IL-6 3′-UTR sequence into pMIR-Report construct (Ambion, Austin, USA). Wild type or mutant IL-6 3′-UTR fragment (from 84 to 127) was amplified and cloned into the luciferase reporter via *Spe* I and *Hind* III sites. All the primers were listed in Table 1. Cells plated in a 96-well plate were co-transfected with 100 nM single-stranded miRNA inhibitors or negative control inhibitors, 50 ng of firefly luciferase reporter and 10 ng of pRL-TK (Promega, USA) using the JetPRIME reagent. Cells were collected 36 h after last transfection and analyzed using Dual-Luciferase Reporter Assay System (Promega).

**Table 1:** **All primers used in this study**

**Name**	**Sequence**
U6 F	5′-CTCGCTTCGGCAGCACA-3′
U6 R	5′-AACGCTTCACGAATTTGCGT-3′
β-actin F	5′-AGAGGGAAATCGTGCGTGAC-3′
β-actin R	5′-CAATAGTGATGACCTGGCCGT-3′
IL-6 (WT) F	5′-AAACTAGTGAAATATATCCTGTTGTCAGG-3′
IL-6 (WT) R	5′-GGAAGCTTCCAACATTCATATTGTCAGTTC-3′
IL-6 (MUT) F	5′-AAACTAGTGAAATATATCCTGTTGTCAGG-3′
IL-6 (MUT) R	5′-GGAAGCTTCCTAGAGTAACATTGTCAGTTC-3′
IL-6 F	5′-AACGATGATGCACTTGCAGA-3′
IL-6 R	5′-GAGCATTGGAAATTGGGGTA-3′
P65 F	5′-ATGGCTACTATGAGGCTGACC-3′
P65 R	5′-GCTGGCTAATGGCTTGCT-3′
pri-miR-181b F	5′-CAGCACATCTCTGCCTCACA-3′
pri-miR-181b R	5′-TTGCGGTTCTGTCTTCAGC-3′
miR-181b RT	5′-GTCGTATCCAGTGCAGGGTCCGAGGTATTCGCACTGGATACGAACCCAC-3′
miR-181b F	5′-AAAACATTCATTGCTGTCG-3′
miR-181a RT	5′-GTCGTATCCAGTGCAGGGTCCGAGGTATTCGCACTGGATACGAACTCAC-3′
miR-181a F	5′-TGAACATTCAACGCTGTCG-3′
miR-181c RT	5′-GTCGTATCCAGTGCAGGGTCCGAGGTATTCGCACTGGATACGAACTCAC-3′
miR-181c F	5′-GTAACATTCAACCTGTCG-3′
let-7 g RT	5′-GTCGTATCCAGTGCAGGGTCCGAGGTATTCGCACTGGATACGAAACTGT-3′
let-7 g F	5′-GATCTGAGGTAGTAGTTTGT-3′
miRNA Universal R	5′-GTGCAGGGTCCGAGGT-3′
ChIP F	5′-TTTCTGGTGGCTCTTGATT-3′
ChIP R	5′-ACCTTTCCTCGGCTTTCTA-3′

*Abbreviations:* *F* forward primer, *R* reverse primer *WT* wild type, *MUT* mutant, *RT*, reverse transcription primer.

### Detection of cytokine production

IL-6 production in the cell supernatants were measured with ELISA Kits (ebioscience) according to the manufacturer’s protocols.

### RNA isolation and real-time quantitative PCR (qPCR)

Total RNA was extracted with TRIzol (Invitrogen). Purified mRNA and miRNAs were detected by qRT-PCR assay using SYBR Green detection chemistry. U6 small RNA was used as an internal control for normalization and quantification of miRNA expression. β-actin was used as an internal control for normalization and quantification of IL-6 expression. All primers were listed in Table 1.

### Oligonucleotides transfection

RNA oligos were chemically synthesized and purified by Genepharma Co. Ltd., (Shanghai, China). The sequence of miR-181b inhibitor was 2′-O-Me- ACC CAC CGA CAG CAA UGA AUG UU. The sequence of negative control inhibitor was 2′-O-Me-UUG UAC UAC ACA AAA GUA CUG. p65 siRNA was 5′-AAG AAG CAC AGA UAC CAC CAA dTdT-3′ and 5′-UUG GUG GUA UCU GUG CUU CUU dTdT-3′. Negative control siRNA was 5′-AAU UCU CCG AAC GUG UCA CdTdT-3′ and 5′-GUG ACA CGU UCG GAG AAU UdTdT-3′. The transfections were performed with INTERFERin reagent. The final concentration of miRNA inhibitors was 100 nM. The final concentration of siRNA was 20 nM.

### Western blot analysis

Proteins were separated on a 12% SDS-PAGE gel and transferred onto a nitrocellulose membrane (Bio-Rad, Hercules, USA). The membrane was blocked with 5% non-fat milk and incubated with anti-p65 antibody (Santa Cruz, CA) or anti-β-actin antibody (Sigma, CA, USA). After being washed extensively, a goat anti-mouse secondary antibody was added to the system. The proteins were detected using ECL reagents (Pierce).

### ChIP assay

ChIP assay was performed as described previously [11]. Antibodies to p65 (Santa Cruz sc-109), and control IgG (Santa Cruz sc-2027) were used at 5 μg per immunoprecipitation. 10 μl sonicated but preimmunoprecipitated DNA from each sample was used as input control. All results were normalized to input in each sample. All primers were listed in Table 1.

### Statistical analysis

All statistical analyses were carried out using the SPSS 16.0 statistical software package. Data was presented as the mean ± S.D. Statistical significance was determined by Student’s *t*-test, with values of *P* < 0.05 considered to be statistically significant.

## Results

### miR-181b and IL-6 are differentially expressed during LPS induced tolerance in RAW264.7 cells

**Figure 1:**
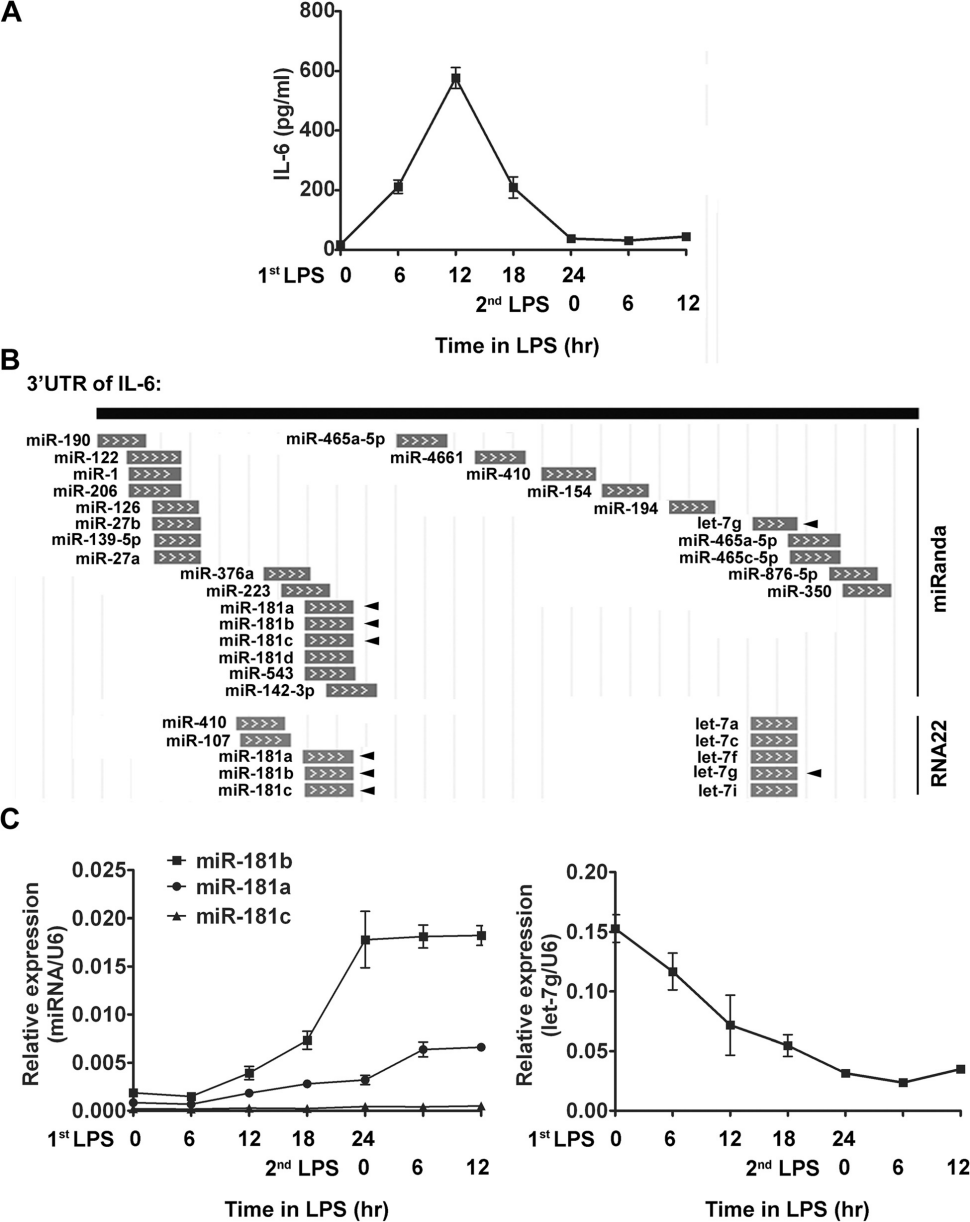
**miR-181b and IL-6 are differentially expressed during LPS induced tolerance in RAW264.7 cells. (A)** RAW264.7 cells were primed with 100 ng/ml LPS (1st LPS) continuously for 24 h and washed twice with PBS, cells were incubated in fresh complete culture medium for 12 h with 100 ng/ml LPS (2nd LPS) for 12 h. Levels of total (intracellular and secreted) IL-6 protein were measured by ELISA. **(B)**. miRNAs targeting 3′-UTR of IL-6 were predicted by miRanda and RNA22. miRNAs predicted by both miRanda and RNA22 were tagged by triangle. **(C)** RAW264.7 cells were treated as in **(A)**. miR-181a, miR-181b, miR-181c and let-7 g were determined by qRT-PCR. Sample data were normalized to U6 mRNA. The samples were assayed in triplicate. Data represent the mean ± S.D. from at least three independent experiments.

Previous study has demonstrated that IL-6 protein significantly increased after LPS stimulation and then incited the “cytokine storm” at the onset of severe systemic inflammation (SSI), and then, IL-6 protein level rapidly decreased and reached background levels by 24 h [12]. This coincided with the induction of LPS tolerance, as cells were unable to respond to a second dose of LPS in RAW264.7 cells (Figure 1A). However, the underlying mechanism which is involved in TLR-triggered IL-6 tolerance remains unclear. In order to investigate potential role of miRNAs in translational repression of IL-6 in LPS induced tolerance. Two commonly used computational target prediction algorithms miRanda (http://www.microrna.org/microrna/home.do) and RNA22 (https://cm.jefferson.edu/rna22v2/) were used to screen the potential miRNAs targeting IL-6. As shown in (Figure 1B), four miRNAs (miR-181a, miR-181b, miR-181c and let-7 g) were predicted by both of these prediction algorithms to have sequences complementary to 3′-UTR of IL-6. Then, we investigated the expression of miR-181a, miR-181b, miR-181c and let-7 g during LPS induced tolerance in RAW264.7 cells. As shown in Figure 1C, miR-181a and miR-181b were increased during first LPS stimulation and maintained these levels during LPS tolerance, while, miR-181c was maintained at a relatively low level and the expression of let-7 g was down-regulated in this process. The most significantly upregulated miR-181b, which could rescue septic mice by regulating the NF-κB signaling pathway [13], was selected for further study.

### IL-6 is the direct target of miR-181b

**Figure 2:**
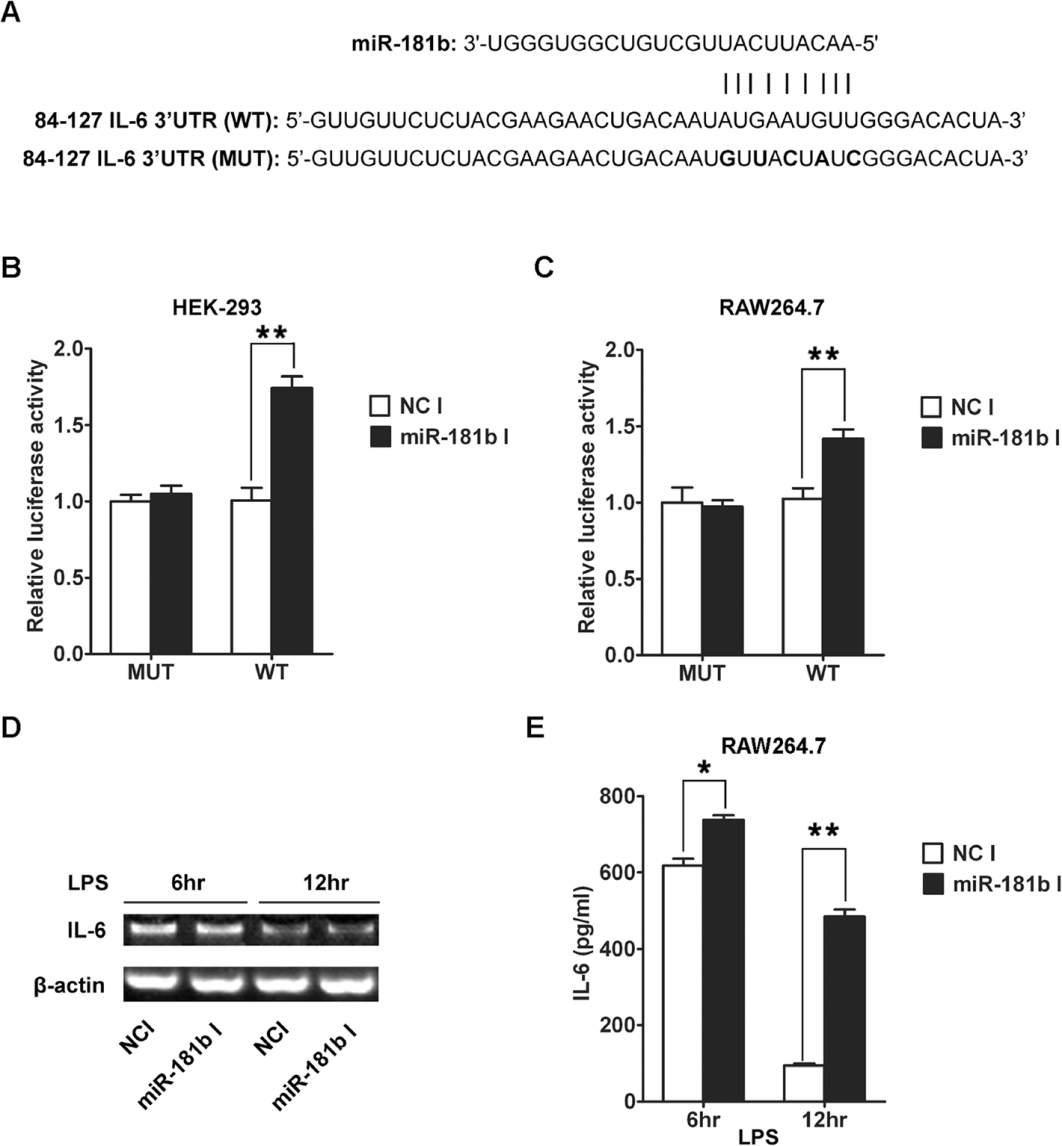
**miR-181b directly targets IL-6 gene. (A)** miR-181b and its putative binding sequence in the 3′-UTR of IL-6. Mutant sequences were shown in bold type. (WT: wild type; MUT: mutant type). **(B, C)** Analysis of luciferase activity. **(B)** HEK-293 cells and **(C)** RAW264.7 cells were cotransfected with miR-181b inhibitors or negative control inhibitors, pRL-TK and firefly luciferase reporter plasmid containing 3′ UTR of IL-6 gene. pRL-TK was cotransfected as an internal control to correct the differences in both transfection and harvest efficiencies. The firefly luciferase activity of each sample was normalized to the Renilla luciferase activity. The normalized luciferase activity of negative control inhibitors was set as relative luciferase activity 1 respectively. **(D)** Effects of miR-181b inhibitors on the endogenous IL-6 mRNA levels. RAW264.7 cells were cotransfected with miR-181b inhibitors or negative control inhibitors. 24 h after transfection, cells were primed with 100 ng/ml LPS continuously for 6 h and 12 h, and then cells were isolated, the expression of IL-6 was analyzed by qPCR. **(E)** Effects of miR-181b inhibitors on the endogenous IL-6 protein levels. 24 h after transfection with the miR-181b inhibitors or negative control inhibitors, cells were primed with 100 ng/ml LPS continuously for 6 h and 12 h, and then cells were isolated. RAW264.7 cells were analyzed by ELISA. NC: negative control; I: inhibitors. The data were subjected to Student’s *t*-test. *p < 0.05, **p < 0.01.

In order to investigate whether miR-181b could target and regulate the expression of IL-6, we performed the following experiments. We found the potential targeting sequence for miR-181b is within the 3′-UTR of IL-6 mRNA from 110 to 118. We first performed luciferase reporter assays in HEK-293 cells. We created luciferase reporter plasmid with wild type or mutant targeting sequence of IL-6 mRNA (Figure 2A), which were cotransfected with miR-181b inhibitors or negative control inhibitors into HEK-293 cells for 48 h, followed by measurement of luciferase activity in transfected cells. Our results showed that the reporter plasmid with wild-type 3′-UTR of IL-6 caused a significant increase in luciferase activity in cells transfected with miR-181b inhibitors, whereas reporter plasmid with mutant 3′-UTR of IL-6 produced no change in luciferase activity (Figure 2B). We further performed luciferase reporter assays in RAW264.7 cells. As shown in Figure 2C, miR-181b inhibitors also markedly increased the luciferase activity in RAW264.7 cells. Then, we explored whether the endogenous IL-6 in RAW264.7 cells was regulated by miR-181b similarly. RAW264.7 cells were transfected with miR-181b inhibitors or negative control inhibitors, 24 h later, these cells were stimulated with 100 ng/ml LPS for 6 h and 12 h, IL-6 mRNA and protein levels were examined by RT-PCR and ELISA, respectively. IL-6 mRNA expression was not affected by these inhibitors (Figure 2D). The level of IL-6 protein was consistently and substantially up-regulated by miR-181b inhibitors (Figure 2E). Taken together, our results demonstrated that IL-6 was a direct target of miR-181b in macrophages.

### Up-regulation of miR-181b during LPS stimulation in RAW264.7 cells is associated with NF-kB

**Figure 3:**
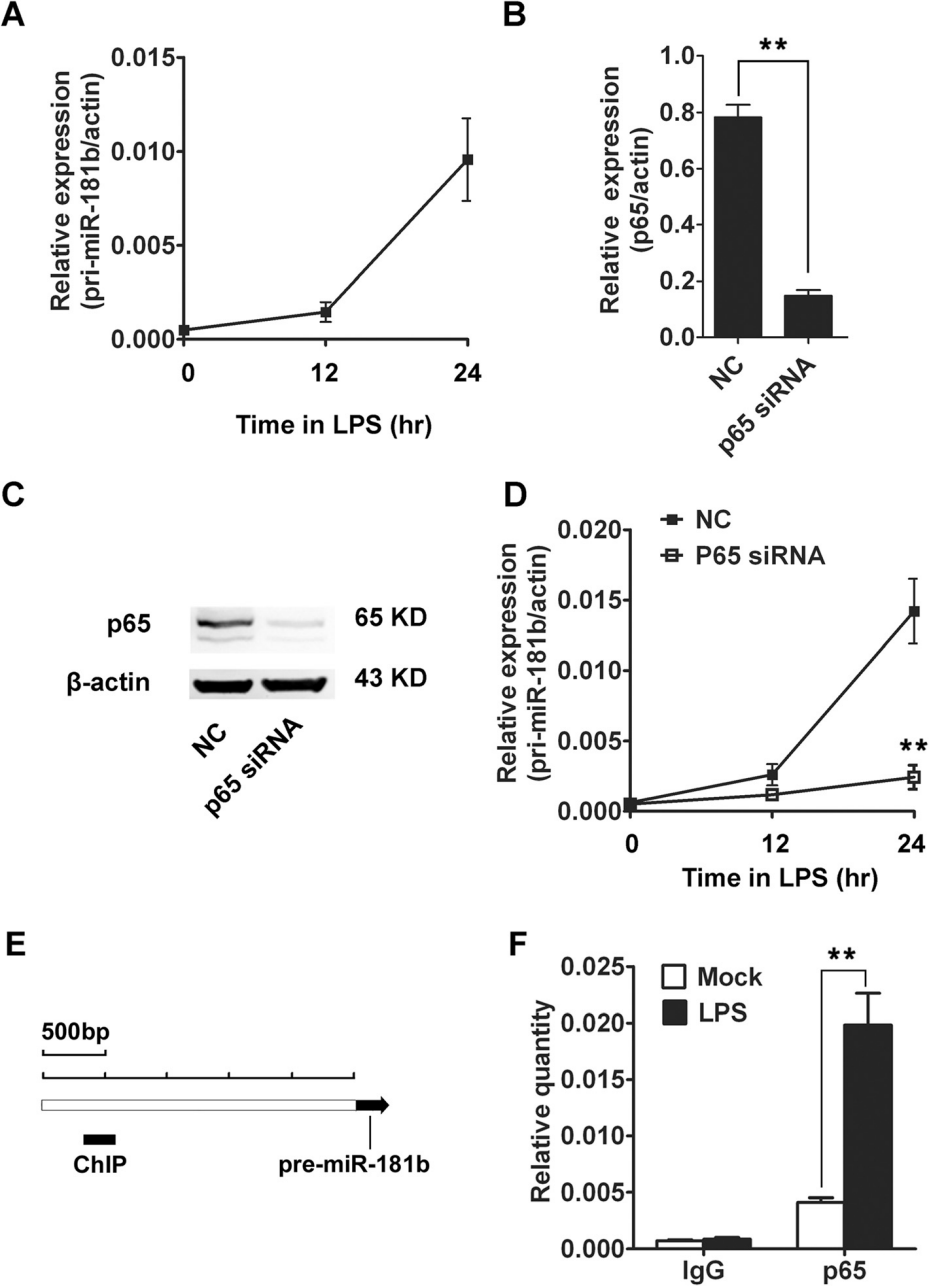
**Up-regulation of miR-181b in LPS tolerance is NF-kB-dependent. (A)** RAW264.7 cells were primed with 100 ng/ml LPS continuously for 12 h and 24 h. pri-miR-181b were determined by qRT-PCR. Sample data were normalized to β-actin mRNA. **(B, C)** qRT-PCR and western blots of p65 in RAW264.7 cells after p65 siRNA or negative control siRNA transfection. β-actin was used as an internal control. **(D)** RAW264.7 cells were transfected with p65 siRNA or negative control siRNA, 24 h later, cells were primed with 100 ng/ml LPS continuously for 12 h and 24 h. pri-miR-181b were determined by qRT-PCR. Sample data were normalized to β-actin mRNA. **(E)** One set of primers was used in the qPCR analysis to span the potential promoter region of miR-181b. **(F)** RAW264.7 cells were primed with 100 ng/ml LPS continuously for 24 h. qPCR analysis of p65 binding in the promoter region of miR-181b by ChIP assay. NC: negative control. The data were subjected to Student’s *t*-test. *p < 0.05, **p < 0.01.

The expression of several miRNAs such as miR-155 and miR-146 has been demonstrated to increased after LPS treatment in previous studies, which have been a clue in LPS response and tolerance models [14]. To investigate the potential mechanism of up-regulation of miR-181b by LPS, firstly, we analyzed the pri-miR-181b expression in RAW264.7 cells after LPS stimulation by qRT-PCR. As shown in Figure 3A, the expression of pri-miR-181b increased during LPS stimulation which was similar to mature miR-181b. These results suggested that up-regulation of miR-181b in LPS induced tolerance was regulated at transcriptional levels. NF-kB was reported to regulate expression of miR-146a at transcriptional levels [15]. So we investigate whether up-regulation of miR-181b might be associated with NF-kB. Knockdown of NF-kB was performed using one siRNA for p65 gene, which was the main complex of NF-kB [16]. The mRNA and protein expression of p65 were detected by qRT-PCR and Western blotting, as shown in Figure. 3B and C, the expression of p65 in RAW264.7 cells was down-regulated significantly by p65 siRNAs. We also detected the expression of pri-miR-181b during LPS stimulation after knockdown of NF-kB, the results showed that up-regulation of pri-miR-181b during LPS stimulation was suppressed significantly in NF-kB knockdown cells (Figure 3D). The regions (2500 bp upstream of pre-miRNA) were postulated as promoters of miRNAs to investigate the epigenetic regulation of miRNA expression [11]. So we chose this region as potential promoter to investigative the p65 binding of miR-181b. ChIP assays showed that p65 binding activity was up-regulated in promoter region of miR-181b during LPS stimulation (Figure 3E, F). Taken together, our results demonstrated that up-regulation of miR-181b in LPS tolerance was NF-kB-dependent.

### LPS-induced down-regulation of IL-6 during tolerance is abrogated by miR-181b inhibitors

**Figure 4:**
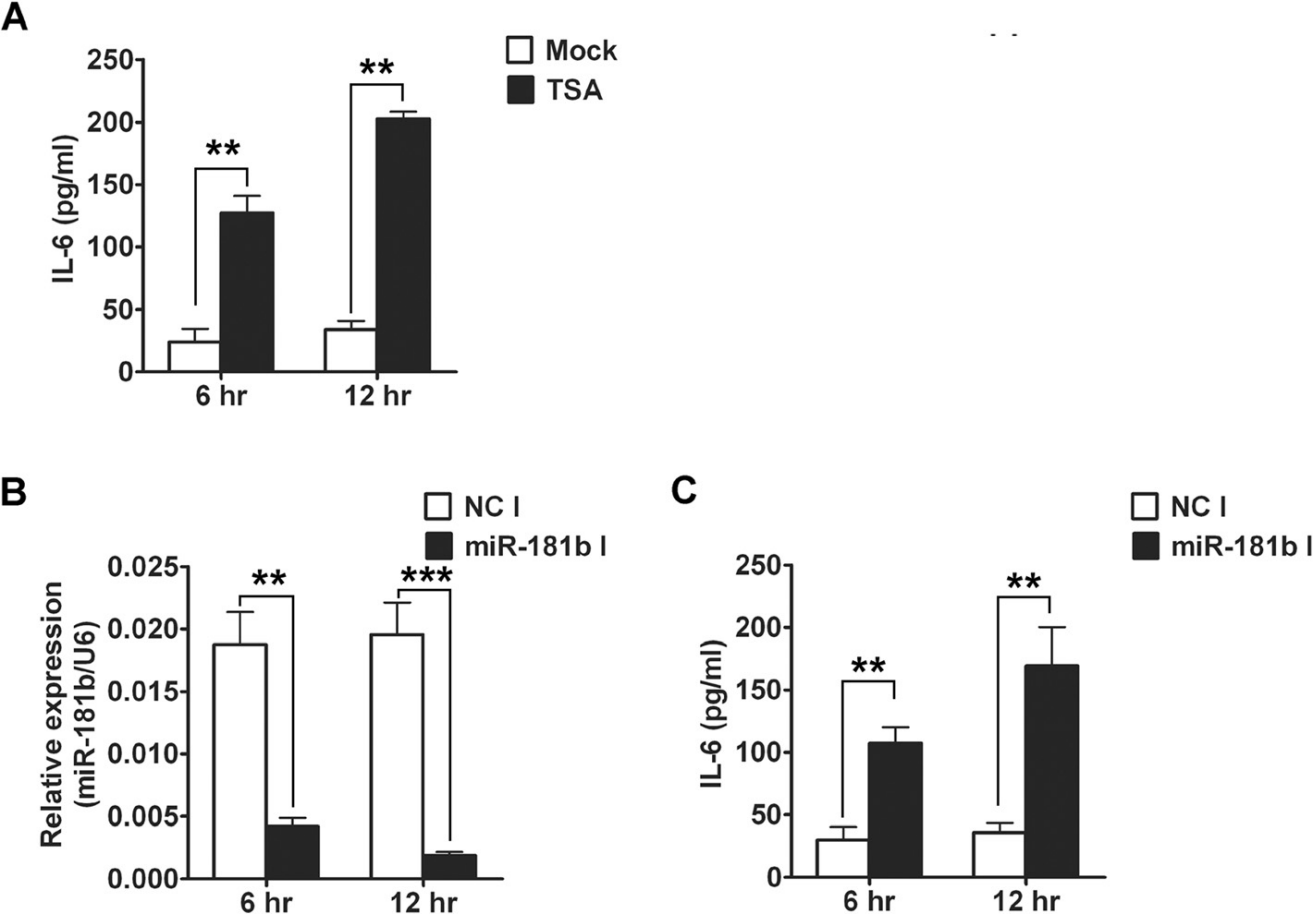
**miR-181b inhibitors abrogates LPS-induced IL-6 tolerance. (A)** Effects of TSA on the endogenous IL-6 protein levels during LPS tolerance. RAW264.7 cells were primed with 100 ng/ml LPS continuously for 24 h and washed twice with PBS, then cells were primed with 1 μM TSA and 100 ng/ml LPS. Another 6 h and 12 h later, IL-6 were analyzed by ELISA. cells were primed with 100 ng/ml LPS continuously for 24 h and washed twice with PBS, then cells were transfected with miR-181b inhibitors and primed with 100 ng/ml LPS. 6 h and 12 h later, miR-181b were analyzed by qRT-PCR **(B)** and IL-6 were analyzed by ELISA **(C).** NC: negative control; I: inhibitors. The data were subjected to Student’s *t*-test. **p < 0.01, ***p < 0.001. **(B, Ccp** Effects of miR-181b inhibitors on the endogenous IL-6 protein levels during LPS tolerance. RAW264.7.

Considering down-regulation of IL-6 acts as a hallmark of endotoxin-tolerant macrophages [14], we further investigated whether LPS-mediated up-regulation of miR-181b was involved in the induction of LPS-hyporesponsiveness. Recently, a few reports have shown that epigenetic silencing contributed to the induction of LPS-hyporesponsiveness [17,18]. When treating macrophages with Trichostatin A (TSA), a specific inhibitor of histone deacetylase (HDAC), the tolerant cells produce IL-6 again [19]. So we firstly investigated the effect of TSA on tolerance reversion of IL-6. RAW264.7 cells were primed with 1st LPS treatment for 24 h, and then followed by TSA treatment and 2nd LPS treatment. Our results showed that TSA up-regulated IL-6 expression in tolerant macrophages (Figure 4A). Nextly, we explored whether miR-181b also up-regulated the expression of IL-6 during LPS induced tolerance. RAW264.7 cells were primed with 100 ng/ml LPS for 24 h, followed by transfection with miR-181b inhibitors, and then followed by secondary LPS challenge. Interestingly, comparing with the cells transfected with negative control inhibitors, RAW264.7 cells transfected with miR-181b inhibitors produced significantly higher levels of IL-6 in response to secondary LPS challenge as shown in Figure 4B, C, which was similar to TSA treatment. These results demonstrated that LPS-mediated up-regulation of miR-181b was, at least in part, involved in the induction of IL-6 tolerance in RAW264.7 cells.

## Discussion

In this study, we detected a significant increase of miR-181b expression in RAW264.7 cells following LPS stimulation, and up-regulation of miR-181b was associated with NF-kB. We also demonstrated that miR-181b targeted the 3′-UTR of IL-6 and down-regulated expression of IL-6 at protein levels. Moreover, up-regulation of miR-181b in response to LPS may be required for inducing IL-6 tolerance in macrophages.

IL-6 is secreted by macrophages and T cells to stimulate immune response during infection or trauma [20]. IL-6 is one of the most important inflammatory factors in fever and the acute phase response. The fine tuning of IL-6 expression is crucial since dysregulation of IL-6 has been attributed to many diseases such as atherosclerosis [21], systemic lupus erythematosus [22], Behçet′s disease [23], diabetes [24], multiple myeloma [25], depression [26], Alzheimer′s Disease [27], prostate cancer [28] and rheumatoid arthritis [29]. However, the mechanism underlying the fine tuning of IL-6 expression especially in infection induced tolerance remains unclear. Herein, we provide a new molecular mechanism to be responsible for IL-6 tolerance in macrophage after LPS stimulation. We found that miR-181b expression levels and IL-6 secretion were inversely correlated after LPS stimulation. We further confirmed that IL-6 3′-UTR contained one miR-181b binding site and LPS induced endotoxin tolerance due to the down-regulation of IL-6 production which was induced by miR-181b overexpression. So, it suggested that miR-181b mediated posttranscriptional control could be involved in fine tuning the critical level of IL-6 expression in endotoxin tolerance. Our results confirmed, in the course of LPS induced tolerance, IL-6 was regulated not only by epigenetic modification at the transcriptional level but also by miRNAs at the post-transcriptional level. Our results were similar to the previous findings in TNF-α [12].

miR-181b belongs to the miR-181 family including four members miR-181a, miR-181b, miR-181c, and miR-181d. The biological functions of miR-181b were widely investigated. miR-181b was defined as a regulator of the B cell primary antibody repertoire based upon its ability to restrict the activity of activation-induced cytidine deaminase [30]. Altered expression levels of miR-181b have been detected in multiple tumors and leukemia/lymphoma [31-35]. Sun et. al identified patients with sepsis having reduced circulating plasma levels for miR-181b compared with control patients without sepsis and identified miR-181b as a cytokine-responsive miRNA which regulates the endothelial response to inflammation by regulating the NF-κB signaling pathway [13]. In our study, we identified miR-181b as a endotoxin-responsive miRNA with a NF-κB-dependent manner during LPS induced IL-6 tolerance in macrophages. These findings coupled with the improved survival observed in miR-181b “rescue” studies in septic mice suggest that therapies directed at restoring miR-181b expression may ameliorate this acute inflammatory process [13]. miR-181 family including miR-181a, miR-181b, miR-181c and miR-181d have the similar sequence. Whether other miR-181 s have the similar function like miR-181b during LPS induced IL-6 tolerance remains to be investigated.

## Conclusions

Based on our results, we showed that macrophages were stimulated by LPS to up-regulate the expression of miR-181b, and this is related to the immune tolerance of IL-6. miR-181b down- regulated the expression of IL-6, thus to avoid the excessive immune response which induced by LPS. We think that we find a target to break the immune tolerance of IL-6 and a new way to control the clinical infectious disease.

## Abbreviations

miRNA: microRNA
3′-UTR: 3′-untranslated region
PRRs: Pattern recognition receptors
TLR: Toll-like receptors
MyD88: Myeloid differentiation primary-response protein 88
LPS: Lipopolysaccharide
IL: Interleukin
TNF-a: Tumor necrosis factor a
TGF-β: Transforming growth factor β
PPAR gamma: Peroxisome proliferator-activated receptor gamma
FBS: Fetal bovine serum
qPCR: real-time quantitative PCR
NC: negative control
